# Identification of Health Risks of Hand, Foot and Mouth Disease in China Using the Geographical Detector Technique

**DOI:** 10.3390/ijerph110303407

**Published:** 2014-03-21

**Authors:** Jixia Huang, Jinfeng Wang, Yanchen Bo, Chengdong Xu, Maogui Hu, Dacang Huang

**Affiliations:** 1 State Key Laboratory of Resources and Environmental Information System, Institute of Geographic Science and Natural Resource Research, Chinese Academy of Sciences, Beijing 100101, China; E-Mails: huangjx@lreis.ac.cn (J.H.); xucd@lreis.ac.cn (C.X.); humg@lreis.ac.cn (M.H.); 2 Key Laboratory of Surveillance and Early Warning on Infectious Disease, Chinese Center for Disease Control and Prevention, Beijing 102206, China; 3 School of Geography, Beijing Normal University, Beijing 100875, China; E-Mail: boyc@bnu.edu.cn; 4 State Key Lab of Remote Sensing Science, Chinese Academy of Science, Beijing 100094, China;; 5 School of Geographical Science, Northeast Normal University, Changchun 130024, China;E-Mail: huangdc@lreis.ac.cn

**Keywords:** hand, foot and mouth disease, geographical detector, interactive effect, industrial structure, population density

## Abstract

Hand, foot and mouth disease (HFMD) is a common infectious disease, causing thousands of deaths among children in China over the past two decades. Environmental risk factors such as meteorological factors, population factors and economic factors may affect the incidence of HFMD. In the current paper, we used a novel model—geographical detector technique to analyze the effect of these factors on the incidence of HFMD in China. We collected HFMD cases from 2,309 counties during May 2008 in China. The monthly cumulative incidence of HFMD was calculated for children aged 0–9 years. Potential risk factors included meteorological factors, economic factors, and population density factors. Four geographical detectors (risk detector, factor detector, ecological detector, and interaction detector) were used to analyze the effects of some potential risk factors on the incidence of HFMD in China. We found that tertiary industry and children exert more influence than first industry and middle school students on the incidence of HFMD. The interactive effect of any two risk factors increases the hazard for HFMD transmission.

## 1. Introduction

Hand, foot and mouth disease (HFMD) is a common infectious disease, which in China mainly occurs among children younger than 5 years old [1]. This disease often starts with a fever, and is then followed by pharyngitis, mouth ulcers, and a rash appearing on the hands and feet [2]. HFMD is caused by viruses, which belong to the genus *Enterovirus*. These viruses include polioviruses, coxsackieviruses, echoviruses, and enteroviruses. By means of saliva, feces, vesicular fluid, respiratory secretions, and respiratory droplets, the virus spreads to other people [2,3]. In recent years, HFMD was prevalent in areas of Southeast Asia, such as Thailand, Taiwan, and Singapore [2,4,5,6] and represented a serious health hazard. In 2008, a serious epidemic of HFMD broke out on the Chinese mainland, and there were over 600,000 clinical cases accompanied by 126 deaths from March 2008 to June 2009. At the center of Anhui province, there were over 6,000 HFMD clinical cases and 22 cases of child death [2,7].

The risk factors and transmission patterns of HFMD have been well studied [5,8,9]. Meteorological factors, including temperature, relative humidity, and rainfall, have a strong influence on the spread of the disease [3,10,11]. Urban areas have a higher incidence of HFMD than rural areas, and cities with a higher population density and stronger population mobility suffer more from HFMD [12]. Some previous studies found that the peak of HFMD appears in late spring and early summer in the Chinese mainland and in summer in Taiwan [5,8]. Research from the UK showed that HFMD has an epidemic period of three years [9].

However, there is little research on the influence of different industrial structures on the incidence of HFMD. Furthermore, most previous studies on HFMD simultaneously analyzed the effect of a single environmental factor on the incidence of HFMD. Therefore, there is a lack of studies on the interactive effect of two or more environmental factors on the incidence of HFMD. Previous research found that population density is an important factor that affects the spread of HFMD [12,13,14], but few studies have estimated the influence of different populations (e.g., children, students, and adults) on the incidence of HFMD in China. HFMD is mainly prevalent among children aged 0–9 years old [11], but the effect of different age groups on the incidence of HFMD is unknown. Whether cities with different industrial structures display different transmission patterns on the spread of HFMD is also unknown.

In this study, we used the geographical detector method [15] to estimate the relationship between the incidence of HFMD and environmental risk factors such as meteorological factors, population factors and economic factors. As a novel spatial analysis method, the geographical detector method does not require any assumptions or restrictions with respect to explanatory and response variables [16]. By means of the concept of the power of determinant (PD), four geographical detectors were applied to estimate the effect of environmental risk factors on the incidence of HFMD. A risk detector was used to calculate the geographical area under environmental health risk. A factor detector was used to assess which determinants are responsible for the incidence of HFMD. An ecological detector determined if there is a significant difference between the effects of different environmental factors on HFMD. An interactive detector was used to analyze whether multi determinants independently or dependently affect the spread of HFMD [15]. All four detectors can be easily implemented using the software Excel-GeogDetector. An excellent feature of the geographical detector method is that it can deal with quantitative and qualitative data, while the classic regression model has some limitations on analyzing qualitative data when there are too many categories [16,17].

## 2. Data and Methods

### 2.1. HFMD Data

The HFMD data used in this research were provided by the Chinese Center for Disease Control and Prevention. The data included the number of children aged 0–9 years suffering from HFMD in each county during May 2008 in China (not including Taiwan). Previous studies have shown that HFMD mainly occurs among children, and the probability of an adult suffering from HFMD is small [2]. In recent years, the prevalence of children with HFMD aged 0–4 years has significantly declined compared with that of children aged 5–9 years [2]. Therefore, we chose children aged 0–9 years as the research population. To reduce the effect of population size, we used the cumulative incidence (CI) to reflect the risk of contracting HFMD in each county. In this study, the crude CI was the ratio of the number of HFMD cases (*N_i_*) aged 0–9 years and the total child population number (*P_i_*) aged 0–9 years. However, the number of HFMD cases may be zero in some counties. Therefore, this may lead to deviation when dividing the cases by the total number of children in the population. We adopted the Hierarchical Bayesian model to reduce the spatial variance of CI [15,18]. We used log (λ*_i_*) to denote the logarithm of the expected CI in the *i^th^* county, and log (λ*_i_*) consists of three parts: the overall level of the disease risk α, the correlated heterogeneity *u_i_* and the uncorrelated heterogeneity *v_i_*, as following equation [11]:

log(λ*_i_*) = α + *u_i_* + *v_i_* (1)

For the correlated heterogeneity *u_i_*, spatial correlation is defined by the intrinsic Gaussian auto-regression model, while the uncorrelated heterogeneity *v_i_* is an independent normal variable with mean 0 and variance 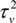, as following equation [18,19].

*V_i_* ~ *N*(0, *τ*_v_^2^) (2)

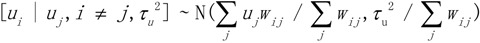
(3)

**Figure 1:**
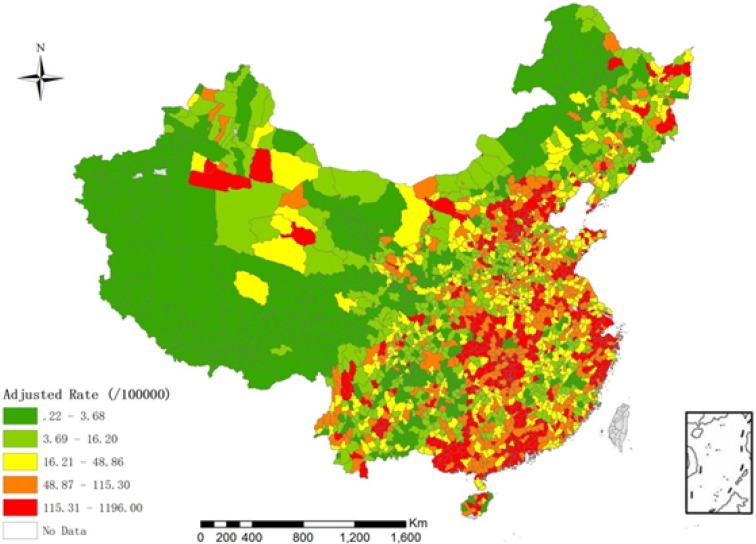
Spatial distribution of the incidence of children with HFMD who were aged between 0 and 9 years old in China in May 2008 after adjustment by the Hierarchical Bayesian model. Red represents a higher incidence of HFMD and green denotes a lower incidence.

*w_ij_* is the spatial adjacent of the connectivity between counties. If the *i^th^* county and *j^th^* county is adjacent, then *w_ij_* = 1, otherwise *w_ij_* = 0. The variabilities of *u_i_* and *v_i_* are denoted by 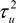 and 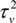, respectively. In this model, we use gamma distribution as prior distribution of the hyper-parameters: 1/*τ*_v_^2^ ~ *Gamma*(0.001, 0.001) and 1/*τ*_u_^2^ ~ *Gamma*(0.5, 0.0005) [11]. The Hierarchical Bayesian model is conducted by MCMC simulation in WinBUGS 1.4, and the length of burn-in sequence is 5,000 [11]. The incidence of children is shown in Figure 1 (after adjustment by the Hierarchical Bayesian model).

### 2.2. Determinants of HFMD and Their Proxies

**Figure 2:**
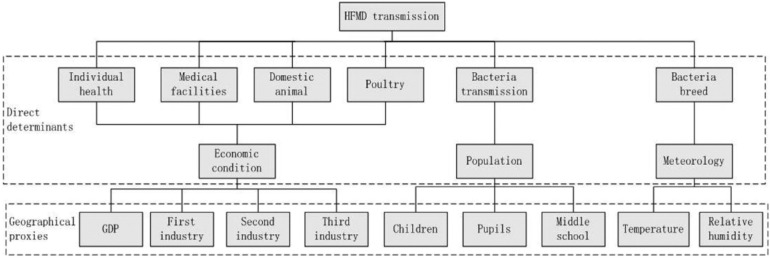
Determinants of HFMD and their proxies.

Because environmental and economic factors affect the incidence of HFMD [3,11,12], we considered both of these factors in our study. One of the most important factors that could affect the transmission of HFMD is the pathogenic bacteria environment, including the bacteria breeding environment and bacteria transmission environment. For the bacteria breeding environment, we selected monthly mean temperature, monthly precipitation, and relative humidity as proxy variables [11]. Because population mobility affects the incidence of HFMD [12], we chose the density of children aged 0–9 years, pupil density, and middle school student density as proxy variables to reflect the bacteria transmission environment. Another important factor that could affect the transmission of HFMD is the source of pathogenic bacteria, including the densities of domestic animal and poultry. In this research, we considered that industry structure could reflect the source of HFMD bacteria. Primary industry includes agriculture, including farming, forestry, animal husbandry, and fisheries. Secondary industry is mainly manufacturing. Tertiary industry is mainly composed of transportation, financial services, and the food and beverage industry. The third factor is individual immunity, including individual health and medical facilities, and this factor may be reflected by global domestic product (GDP). The proxy variable associations of potential factors that could affect the incidence of HFMD are shown in Figure 2.

Meteorological data were collected from the China Meteorological Data Sharing Service System. There are a total of 727 meteorological stations distributed in China, including three factors that are measured: monthly mean temperature, monthly precipitation, and relative humidity. By comparing the average interpolation precision of inverse distance weighting, kriging, and thin plate smoothing splines, we chose the thin plate smoothing spline to estimate each county’s climate factors from the 727 meteorological observation sites [11,20].

Population and economic data were collected from the China Statistical Yearbook for Social and Economic Data of Chinese Counties, the China Statistical Yearbook for Regional Economy, and the China Statistical Yearbook for Cities [21,22,23], including child population aged 0–9 years, pupil population, middle school student population, GDP, first industry, secondary industry, and tertiary industry in each county of 2,309 counties. Population density was calculated by dividing the population of the county by the area of the county.

### 2.3. Geographical Detector

**Figure 3:**
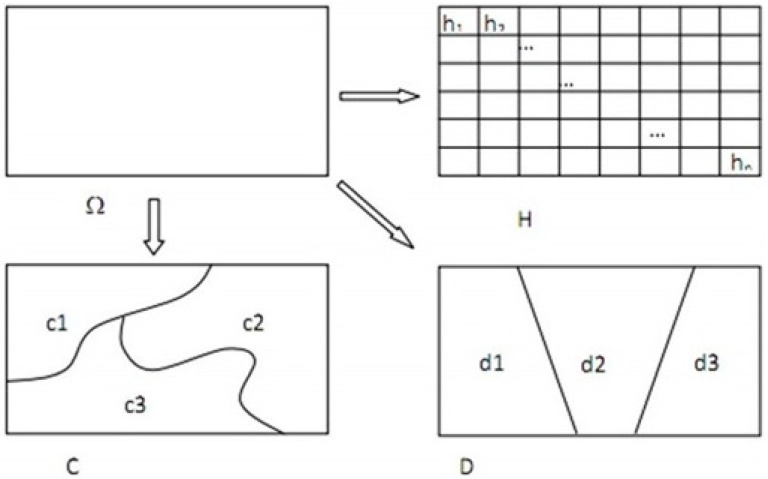
Distribution of the incidence of HFMD (H) and spatial patterns of potential factors C and D in the study area [15].

We assumed that if HFMD was influenced by a particular factor, then the distribution of this factor would be similar to that of the disease in geographical space [15]. This has two meanings: the first one is that the risk factor may positively influence the incidence of HFMD, and the second one is that the spatial distribution of risk factor has a negative relationship with that of the incidence of HFMD. In our study, if some factor had an effect on the incidence of HFMD, then the spatial distribution of this factor and the distribution of the incidence of HFMD may be more consistent (Figure 3).

Geographical space was denoted as Ω and the spatial distribution of the incidence of HFMD was represented as *H*. The whole geographical space was divided by regular grids into *N_T_* units, and the incidence of HFMD in each unit was denoted as *H_i_* (1 ≤ *i* ≤ *N_T_*). A risk factor, which may influence the incidence of HFMD, was denoted as *D* in the space, and this factor was divided into *n_D_* sub-regions in the geographical space. After intersecting the disease (*H*) and the risk factor (*D*), there were *n_D_* sub-regions in the whole geographical space. Every sub-region had *n_D,Z_* (1 ≤ *z* ≤ *n_D_*) grids and 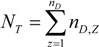. The incidence of HFMD in every grid in the sub-region was defined as *H*_z*,i*_ (1 ≤ *z* ≤ *n_D_*, 1 ≤ *i* ≤ *n_D,Z_*).

The average incidence of HFMD in the entire geographical space Ω was:

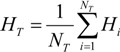
(4)

The sample variance of HFMD in the whole geographical space Ω was:

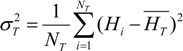
(5)

#### 2.3.1. Risk Detector

The geographical space was divided by the risk factor *D* into several sub-regions, and we denoted two of the sub-regions as *z*_1_ and *z*_2_, respectively. The average incidence of the two sub-regions should be:

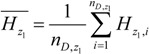
(6)

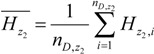
(7)

The sample variances of the incidence of HFMD in the two sub-regions were:

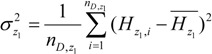
(8)

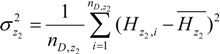
(9)

After calculating the average incidence in each sub-region by formula (3), the seriousness of the HFMD epidemic could be represented as *H*_*z*_1__, *H*_*z*_2__. If there are differences between *H*_*z*_1__ and *H*_*z*_2__, then the incidence of HFMD in these two sub-regions may be different. We then determined whether the differences between *H*_*z*_1__ and *H*_*z*_2__ were significant using the t-test (Equation (10) [15]):

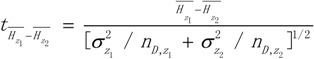
(10)
The degree of freedom was:

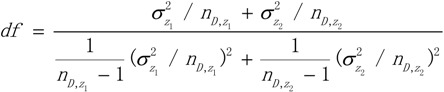
(11)

To test the null hypothesis *H*_0_ : *H*_*z*_1__ = *H*_*z*_2__, we used the confidence level α (generally 5%). If 
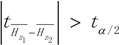
, we can then reject *H*_0_, denoting that the incidence of HFMD in these two sub-regions was significantly different; otherwise the difference between them may be caused by error.

#### 2.3.2. Factor Detector

The risk factor *D* divided the geographical space Ω into several sub-regions, and we could then calculate the overall variance caused by the risk factor *D*:

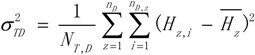
(12)
where 
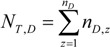
. If the risk factor affected the incidence of HFMD completely, then the sample variance in each sub-region was equal to 0 [15], which means that 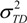 was also equal to 0. We defined the power of determinant (PD) as:

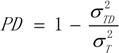
(13)

The PD value is between 0 and 1. When the PD value approaches 1, the value of 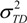 is close to 0, which means that this factor has the same distribution as the incidence of HFMD.

#### 2.3.3. Ecological Detector

We used the ecological detector to analyze which factors influence HFMD more than another factor, and whether the differences between them are significant. Using temperature and humidity as an example, we wished to determine which factor affected the incidence of HFMD the most. Assuming that the two factors are *D*_1_ and *D*_2_, the overall variance of these two risk factors is 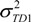 and 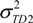, respectively. The total number of grids divided by these risk factors is *n*_*T,D*1_ and *n*_*T,D*2_ respectively. To compare the differences between 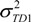 and 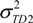, the F test was used:

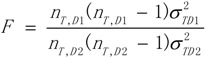
(14)

This statistical model approximately obeys *F*(*n_T,D_*_1_ − 1, *n_T,D_*_2_ − 1) distribution, and the degree of freedom is *df* = (*n_T,D_*_1_, *n_T,D_*_2_) [24]. To test the null hypothesis 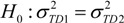, the confidence level was calculated (generally α = 5%). If *H*_0_ was rejected under the confidence level α, this indicated that these two factors had a significant difference on the influence of HFMD.

#### 2.3.4. Interactive Detector

We used the interactive detector to analyze the effect of the interaction of two or multiple factors on HFMD. Two risk factors, *D*_1_ and *D*_2_, may be independent or have a combined effect on HFMD. If there is a combined effect, the effect of these factors on HFMD will be greater after intersecting. We used GIS software to stack the geographical layers *D*_1_ and *D*_2_, and obtained a new geographical layer *E*. By comparing the value of PD of *D*_1_, *D*_2_ and *E*, we were able to determine the influence of the intersection [15,16]. All four detectors were easily implemented using the new software Excel-GeogDetector, which can be freely downloaded at http://www.sssampling.org/Excel-GeoDetector.

## 3. Results

**Figure 4:**
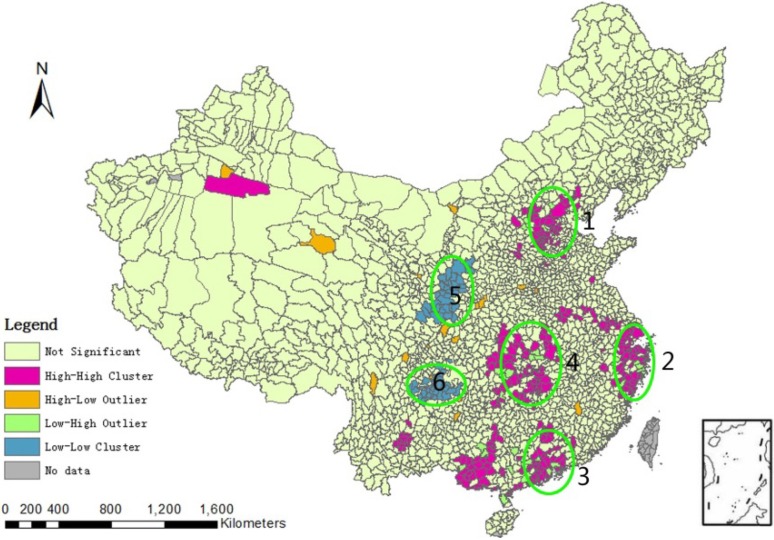
Local Moran’s I of the incidence of HFMD in China.

The spatial heterogeneity of the incidence of HFMD in China was large. Among the 2,309 counties, there were no cases of HFMD in 449 counties (crude CI = 0). To reduce the uncertainty of the crude CI, we used the Hierarchical Bayesian model to adjust for the crude CI [18]. After adjusting for the crude CI, the incidence of HFMD ranged from 0.2/100,000 to 1,196.0/100,000, and the average incidence was 76.1/100,000 (Table 1). The global Moran’s I of the incidence of HFMD was 0.19 (*p* < 0.01), which indicated a weak spatial positive correlation. The local Moran’s I was used to analyze the local clusters of the incidence of HFMD (Figure 4). From the figure, we found that the high-high clusters were Beijing-Tianjin (region 1), the Yangtze River Delta (region 2), the Pearl River Delta (region 3) and the central part of Hunan and Hubei areas (region 4), and the low-low clusters were Shanxi province (region 5) and Sichuan province (region 6).

**Table 1:** Distribution of the incidence of HFMD, meteorological factors, population densities, and economic variables: AT: average temperature; RH: relative humidity; PD0_9: aged 0–9 years density; PupD: pupil density; MSD: middle school student density.

Variables	Mean	Min	25%	50%	75%	Max
Incidence (cases/10^5^)	76.1	0.2	5.3	29.5	94.2	1,196.0
AT (℃)	19.7	−5.2	16.9	21.0	23.1	27.2
Rainfall (mm)	99.6	0.1	38.9	83.7	135.8	497.9
RH (%)	61.3	0.5	52.9	64.4	72.4	91.6
PD0_9 (person/ km^2^)	39.6	0.3	8.9	23.5	55.5	526.2
PupD (person/ km^2^)	29.1	0.1	6.9	17.7	37.9	468.5
MSD (person/ km^2^)	23.2	0.1	4.9	13.1	30.1	514.4
GDP (10^8^ CNY)	153.6	0.8	22.9	55.1	115.4	15,541.0
FirstIndustry (10^8^ CNY)	14.2	0.4	5.1	10.3	19.6	263.7
SecondIndustry (10^8^ CNY)	74.5	1.5	8.0	23.1	58.9	6,167.3
ThirdIndustry (10^8^ CNY)	57.2	0.6	7.2	16.5	35.1	7,599.3

**Figure 5:**
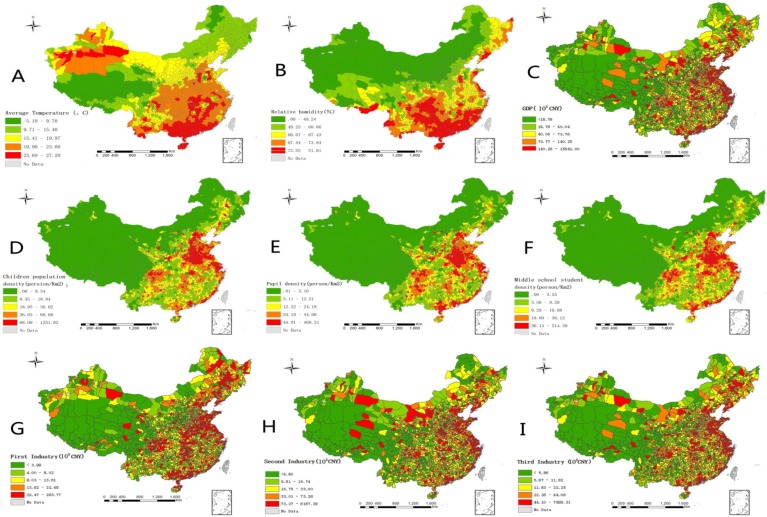
Maps of meteorological factors, population densities, and economic factors of the incidence of HFMD in China. A: Average temperature; B: Relative humidity; C: GDP; D: Child density; E: Pupil density; F: Middle school student density; G: First industry; H: Secondary industry; I: Tertiary industry.

The distribution of meteorological factors, population density, and economic factors significantly varied across China (Table 1). The maximum and minimum values of monthly mean temperature were greatly different. In some counties, the monthly mean temperature was less than 0 °C, while in other counties, it reached 27 °C. The temperature in the southern part of China and west Xinjiang was high, while the temperature in the Tibetan plateau and northeastern area was lower (Figure 5A). The difference in monthly precipitation between areas was also large, with some areas receiving less than 1 mm, but in other areas, it was close to 500 mm. Relative humidity and monthly precipitation were consistent in geographical space, with both of them becoming lower from south to north and east to west (Figure 5B,C). Child density (aged 0–9 years), pupil density, and middle school student density were 39.6 persons/km^2^, 29.1 persons/km^2^, and 23.2 persons/km^2^, respectively (Table 1). The spatial distribution of these three population densities was greatly different. In some counties, the minimal population density was less than 1 person/km^2^; while in other counties, it was more than 500 persons/km^2^. Regions with a higher population density were mainly distributed in Henan, the Yangtze River Delta, and the Pearl River Delta. However, in western and northern China, the population density was lower than other areas (Figure 5D–F). All counties within China have an average GDP of 15.36 billion Yuan, with a minimum of only 0.08 billion Yuan and a maximum of 1,554.1 billion Yuan. This indicates that the gap between the rich and the poor is large in China. Areas with a higher GDP were mainly located in the area of Beijing, the Yangtze River Delta, and the Pearl River Delta (Figure 5G). The average values of first industry, secondary industry, and tertiary industry were 1.42 billion Yuan, 7.45 billion Yuan, and 5.72 billion Yuan respectively. This finding indicated that that secondary industry comprised a maximum proportion of the economy in China. Areas with a strong first industry were mainly located in Sichuan, Shandong, Henan, and northeastern China (Figure 5H), while the coastal areas had developed secondary industry and tertiary industry, and they became less prevalent from east China to west China (Figure 5I,J).

Pearson correlation coefficients were used to test the correlation of the potential variables. Pearson’s correlation coefficient between relative humidity and rainfall was 0.69, and it was 0.89 between secondary industry and tertiary industry. Pearson’s correlation coefficient between pupil density and middle school student density was 0.92, and that between pupil density and child density was 0.82. This correlation coefficient was lower among the other variables than those mentioned above. Therefore, we decided to choose monthly average temperature, relative humidity, GDP, first industry, tertiary industry, child density, and middle school student density as explanatory variables.

We used the risk detector to analyze the effect of different variables on the incidence of HFMD. Table 2 shows the effect of monthly mean temperature on the incidence of HFMD. When the temperature was high, the incidence of HFMD was also high. When temperature was higher than 24 °C, the incidence of HFMD was 122.6/100,000 people. When temperature was less than 7 °C, the incidence of HFMD was only 2.9/10,000 people. This finding indicates that there is a correlation between monthly mean temperature and the incidence of HFMD. As the population density of children aged 0–9 years increased, the incidence of HFMD gradually increased (Table 3). This suggests that there is a correlation between the incidence of HFMD and the population density of children 0–9 years old. Similar analysis was undertaken to analyze the correlation between other variables and the incidence of HFMD using the risk detector.

**Table 2:** Average incidence of HFMD according to the average temperature stratum.

**Stratum**	**<7**	**7–13**	**13–16**	**16–18**	**18–22**	**22–24**	**>24**
Incidence	2.9	18.2	18.6	53.5	51.6	62.8	122.6

Note: Incidence unit: 1/10^5^; average temperature: °C.

**Table 3:** Average incidence of HFMD according to the density stratum of children aged 0–9 years.

**Stratum**	**<0.5**	**0.5–2.5**	**2.5–6.7**	**6.7–26.3**	**26.3–65.8**	**65.8–105.8**	**>105.8**
Incidence	12.4	16.5	42.5	60.3	84.1	130.5	182.8

Note: Incidence unit: 1/10^5^; 0–9 population density: 10^4^ person/km^2^.

We used the factor detector to determine the effect of risk factors on the incidence of HFMD, and this was ranked by PD value as follows: density of children aged 0–9 years (0.25) > tertiary industry (0.23) > GDP (0.20) > middle school student density (0.13) > relative humidity (0.12) > average temperature (0.11) > first industry (0.05). This result showed that the density of children aged 0–9 years could predominantly explain spatial variability of the incidence of HFMD, followed by tertiary industry and GDP, while tertiary industry had a weak influence.

The ecological detector showed that the differences in PD values between child density, tertiary industry, and GDP were not significant (Table 4). In addition, the differences in PD values between average temperature, relative humidity, middle school student density, and first industry were also not significant. However, the differences in PD values between child density, tertiary industry, and GDP were significantly different compared with the other four risk factors. Using the factor detector and ecological detector, we found that child density, tertiary industry, and GDP had a strong effect on the incidence of HFMD.

**Table 4:** Statistical significance of all variables using the ecological detector.

Risk factors	AT	RH	GDP	FI	TI	PD0_9
RH	N					
GDP	Y	Y				
FI	N	N	Y			
TI	Y	Y	N	Y		
PD0_9	Y	Y	N	Y	N	
MSD	N	N	Y	Y	Y	Y

Note: AT: average temperature; RH: relative humidity; GDP: gross domestic product;FI: first industry; TI: tertiary industry; PD0_9: aged 0–9 population density; MSD: middle school density.

The interactive detector showed some interesting phenomena (Table 5). The interactive PD value of tertiary industry and child population density was 0.42, that of GDP and tertiary industry was 0.34, that of child population density and GDP was 0.35, and that of average temperature and relative humidity was 0.28. All of these interactive PD values appeared to be higher than any PD value of sole risk factors. The combinations of the above-mentioned risk factors could effectively explain spatial variability of the incidence of HFMD in China. There were other interactive effects that did not increase the PD value, such as the combination of child population density with middle school student density (0.26).

**Table 5:** PD values for interactions between pairs of factors on the incidence of HFMD.

Risk factors	AT	RH	GDP	FI	TI	PD0_9
RH	0.28					
GDP	0.31	0.28				
FI	0.17	0.16	0.24			
TI	0.31	0.32	0.34	0.26		
PD0_9	0.31	0.33	0.35	0.30	0.42	
MSD	0.23	0.21	0.29	0.19	0.26	0.27

Note: AT: average temperature; RH: relative humidity; GDP: gross domestic product; FI: first industry; TI: tertiary industry; PD0_9: aged 0–9 population density; MSD: middle school density.

## 4. Discussion

In this study, four geographical detectors were used to estimate the effect of economic factors, population densities, and meteorological factors on the incidence of HFMD in China. To our knowledge, this is the first study on the effect of different industries and different populations on the incidence of HFMD in China.

We wished to determine which environmental factor has the greatest role in the transmission of HFMD. Previous studies have shown that temperature, relative humidity, and precipitation have a strong influence on the spread of HFMD [3,10,11]. The household setting, as well as schools and communities, play important roles in the transmission of HFMD [8,25]. There is a clear seasonal pattern for HFMD outbreaks, with a peak in late spring and early summer [5,8,26]. Our study showed that GDP played the greatest role in the incidence of HFMD. Furthermore, we demonstrated that different population densities and industries had different effects on the incidence of HFMD. In addition, the combination of two environment factors strengthened the risk of the incidence of HFMD, especially for the combination of precipitation and temperature. Most previous studies simultaneously considered the influence of a single environmental factor on HFMD transmission. However, the causes of dissemination of HFMD are complex. Our study showed that the interactive detector could simultaneously estimate the effect of two or even more factors on the incidence of HFMD.

We found that children aged 0–9 years had a greater impact on HFMD transmission compared with middle school students. The factor detector showed that the PD value of children aged 0–9 years was 0.25, and that of middle school students was 0.13. Because adult data were not available, we did not analyze the difference in incidence of HFMD between children and adults. Some reports found that children in kindergarten are more easily infected with HFMD [27,28]. Therefore, we believe that children may play a greater role than other populations in the spread of HFMD. There may be two reasons for this phenomenon. The first reason is that children are less likely than adults to have antibodies to protect them, and such antibodies are generated in the body when a person is first exposed to enteroviruses [3]. The second reason is that intimate contact among children, especially in kindergarten, leads to an increased prevalence of HFMD. In the past few years, the morbidity of children aged between 0 and 4 years old has significantly declined compared with that of children between 5 and 9 years old [2]. This study further confirmed that children between 0 and 9 years old have a greater impact on HFMD than middle school students. In the foreseeable years, the size and number of baby care centers and child care centers will significantly increase. This conclusion is supported by the fact that there were 653 childcare centers providing approximately 54,000 places in 2003, and there were 739 childcare centers providing almost 63,000 places at the end of 2007 [2,29,30]. Frequent contact between children will lead to the acceleration of the spread of HFMD, and children will transmit this disease to their family members and other people [2]. Therefore, an important issue for preventing HFMD in the future is to efficiently control its spread among children.

We found that tertiary industry had a greater impact on HFMD than first industry. The factor detector showed that the PD value of tertiary industry was 0.23, while that of first industry was only 0.05. Because tertiary industry is characterized by stronger population mobility, this will lead to the rapid spread of a virus from person to person, finally causing a faster spread of HFMD [12]. However, first industry is mainly constituted by agriculture. People who work in farmland have weak mobility compared with the service industry, and this has decreased the routes of transmission. We did not analyze the effect of secondary industry on the incidence of HFMD for two reasons. First, industry data have shown that there is strong collinearity between secondary industry and the other two industries. Second, secondary industry is mainly composed of manufacturing, and population mobility in secondary industry is between tertiary industry and first industry [31]. Therefore, it is more meaningful to compare the influence of first industry and tertiary industry on the incidence of HFMD.

Our study showed that meteorological factors have a certain effect on the incidence of HFMD. Wang et al. also used the geographical detector technique to explore the relationships between meteorological factors and HFMD transmission [32]. They found that the PD values of mean temperature ranged from 0.02 to 0.11, and the PD values of rainfall ranged from 0.01 to 0.06. Hii et al. found that precipitation has a 1–2-week lag effect on the incidence of HFMD [33]. Wang et al. used the S-BME spatial-temporal model and found that there is a strong relationship between the incidence of HFMD and monthly rainfall [3]. Our study found that the PD values of relative humidity and mean temperature were 0.12 and 0.11, respectively, which is similar to previous results [32]. In our study, the PD value of a single meteorological factor was not high. However, using the interactive detector, we found that the interaction of relative humidity (0.12) and average temperature (0.11) nonlinearly enhanced the incidence of HFMD, which was 0.28. The reason for this finding is because when proper temperature is combined with relative humidity, a virus is more able to survive, reproduce, and transmit [34].

There are some limitations to our study. The first limitation is the discretization problems of quantitative data. For qualitative data, the geographical detector can analyze it effectively, while for quantitative data, it first needs to be discretized and classified into different levels [35]. Our study compared the natural break, quantile break, and equal interval break, and showed no significant difference between them. Therefore we finally chose the natural break to discretize the quantitative data. The problem of how to effectively discretize quantitative data needs to be solved in the future [15,35]. The second limitation is that the time span of the data was only 1 month. Because of this short period, the number of HFMD cases in many counties was 0, and we used the Hierarchical Bayesian model to adjust the morbidity. We chose May 2008, but not the full year, as the study period for two main reasons. The first reason is that HFMD mainly occurs at the end of spring and the beginning of summer in China. Therefore, this period could best represent the trend in incidence of HFMD in China. The second reason is that if we calculated the average value of the data throughout the year, the spatial heterogeneity of the incidence of HFMD would be weak [11]. The third limitation is modifiable area unit problem (MAUP) of geographical detector technique. We chose county scale in this research for two main reasons. The first reason is that HFMD cases were collected from municipal districts and counties. The second reason is that county scale is smaller than province scale and prefecture-level city scale, so the analysis in county scale may reflect the local area characteristics of incidence of HFMD more than other two scales. In the future, we intend to further study MAUP in geographical detector technique.

Although there are some deficiencies in this study, we believe that the geographical detector is an effective statistical method to analyze environmental health risks. Compared with the characteristics of other spatial analytical methods [36,37,38], there may be two main advantages of geographical detector. First, it can effectively analyze the impact of qualitative data (e.g., physiognomy and water systems) on disease. Many statistical models can effectively analyze the influence of quantitative data on dependent variables, but few models can be used to analyze the influence of qualitative data on independent variables. Second, most of the existing statistical models analyze the effect of a single variable on dependent variables in geographical space. However, few models can study the influence of interaction of two or more variables on dependent variables. The geographical detector can also be used to solve the problem of the influence of intersection of multiple variables. In this study, we only analyzed the interactive influence of two factors on HFMD. In the future, we intend to study the common effect of multiple factors on HFMD [11,15].

## 5. Conclusions

This study analyzed the distribution of the incidence of HFMD in mainland China in May 2008. Our data show that HFMD is serious in China. We further used the geographical detector technique to analyze the effect of potential risk factors on the incidence of HFMD in China and found some interesting results. The results of traditional models all show that population density greatly affects HFMD. Our study shows that child density affects the incidence of HFMD more than middle school student density. We also found that tertiary industry plays a greater role in the incidence of HFMD than first industry. Although the PD value of relative humidity is small, once it is combined with temperature, it significantly enhances its influence on HFMD. Our results should be useful for providing instructions and recommendations for the government on epidemic risk responses to HFMD. When HFMD is spreading at a rapid speed, we need to adopt isolation measures to avoid interactive infection of HFMD among children.
